# Suicide risk among residents in a cross-sectional study: the role of the Interpersonal Psychological Theory of Suicide

**DOI:** 10.1192/j.eurpsy.2023.1059

**Published:** 2023-07-19

**Authors:** S. Magliocca, M. Pontiggia, F. Madeddu, R. Calati, P. Zeppegno, C. Gramaglia

**Affiliations:** 1 Department of Psychology, University of Milan-Bicocca, Milan, Italy; 2 Department of Adult Psychiatry, Nîmes University Hospital, Nîmes, France; 3 Institute of Psychiatry, Università del Piemonte Orientale; 4 S.C. Psichiatria, Azienda Ospedaliero Universitaria Maggiore della Carità, Novara, Italy

## Abstract

**Introduction:**

The peculiar requests of postgraduate teaching could affect the students’ lives, predisposing them to mental disorders and suicide risk (e.g. Abreu et al., 2021). The Interpersonal Psychological Theory of Suicide (IPTS) (Joiner, 2005) is a model that has proved useful in explaining this risk.

**Objectives:**

We analyzed risk factors associated with current suicidal ideation (SI) and history of suicidal planning and/or suicide attempt (SP/SA) in a sample of 97 Italian residents in psychological (n= 17, 17.5%) and medical and health care area (n=80, 82.5%) (mean age 29.18±3.25 SD).

**Methods:**

Socio-demographic, psychological (i.e. State-Trait Anxiety Inventory, Beck Depression Inventory – II; BDI-II, Rosenberg Self-Esteem Scale, Reasons For Living Inventory; RFL, Psychache Scale, Mental pain questionnaire; MPQ, Visual Analogue Scale - VAS - on mental pain, Acquired Capability for Suicide Scale-Fearlessness About Death) psychosocial (i.e. Interpersonal Needs Questionnaire; INQ, UCLA Loneliness Scale Version 3, Multidimensional Scale of Perceived Social Support; MSPSS) and somatic pain features (i.e. VAS, Pain Vigilance and Awareness Questionnaire; Self-Awareness Questionnaire; SAQ) were collected through an online questionnaire. We compared residents with SI vs No SI and residents with SP/SA vs No SP/SA and the emerged significant variables, have been inserted in logistic regression models with stepwise method, backward elimination.

**Results:**

The presence of depression (BDI-II), low reasons for living (RFL), psychological pain (Psychache Scale and MPQ), interoceptive awareness (SAQ), Thwarted Belongingness (TB, INQ subscale), loneliness (UCLA) and low perceived social support (MSPSS) were associated with both current SI and history of SP/SA. Concerning regression models, TB (INQ) increased the likelihood of SI while Survival and Coping Beliefs (RFL subscale) reduced it. The model explained 49% of the variance of SI by correctly predicting 88.7% of SI cases. Perceived social support (MSPSS)reduced the likelihood of current SP/SA levels while interoceptive awareness (SAQ) increased it. This model explained 40% of the variance of SP/SA by correctly predicting 82.5% of suicide risk cases.

**Conclusions:**

We have identified risk and protective factors for suicide, consistent with the IPTS, which can orient the prevention, evaluation and clinical treatment of residents.

**Disclosure of Interest:**

None Declared

